# Tumor-Associated Macrophages: A Potential Target for Cancer Therapy

**DOI:** 10.3389/fonc.2021.693517

**Published:** 2021-06-10

**Authors:** Yifan Tan, Min Wang, Yang Zhang, Shengyang Ge, Fan Zhong, Guowei Xia, Chuanyu Sun

**Affiliations:** ^1^ Department of Urology, Huashan Hospital, Fudan University, Shanghai, China; ^2^ Department of Urology, Renmin Hospital of Wuhan University, Wuhan, China; ^3^ Department of Systems Biology for Medicine, Institutes of Biomedical Sciences, Shanghai Medical College, Fudan University, Shanghai, China

**Keywords:** tumor-associated macrophages, tumor microenvironment, cancer immunotherapy, macrophages, cancer

## Abstract

Macrophages, an important class of innate immune cells that maintain body homeostasis and ward off foreign pathogens, exhibit a high degree of plasticity and play a supportive role in different tissues and organs. Thus, dysfunction of macrophages may contribute to advancement of several diseases, including cancer. Macrophages within the tumor microenvironment are known as tumor-associated macrophages (TAMs), which typically promote cancer cell initiation and proliferation, accelerate angiogenesis, and tame anti-tumor immunity to promote tumor progression and metastasis. Massive infiltration of TAMs or enrichment of TAM-related markers usually indicates cancer progression and a poor prognosis, and consequently tumor immunotherapies targeting TAMs have gained significant attention. Here, we review the interaction between TAMs and cancer cells, discuss the origin, differentiation and phenotype of TAMs, and highlight the role of TAMs in pro-cancer functions such as tumor initiation and development, invasive metastasis, and immunosuppression. Finally, we review therapies targeting TAMs, which are very promising therapeutic strategies for malignant tumors.

## Introduction

Cancer is an important public health problem and the second leading cause of death, with approximately 14.1×10^6^ new cancer cases and 8.2×10^6^ deaths worldwide each year (1). Lung, breast and colorectal cancers are the most commonly diagnosed neoplasms, while the top three leading causes of death are from malignancy of the lung, liver and stomach (1, 2). Although advances in treatment strategies have resulted in an increase in overall survival rates for many cancers, some patients may experience recurrence or even distant metastases, leading to advanced stages of the disease (3). Thus, a better understanding of tumor biology is critical for the development of novel therapeutic strategies for cancer patients. The pathogenesis of cancer is intricate, involving not only alterations in the genomes of tumor cells, but also in the microenvironment in which they reside (4). The tumor microenvironment (TME), containing immune cells (e.g., neutrophils, macrophages, T cells etc.), fibroblasts, endothelial cells, secreted molecules and extracellular matrix, plays an important role in promoting tumor progression and has therapeutic potential (5). Among these cells, macrophages are a prominent component of the tumor microenvironment, representing an evolutionarily ancient cell type involved in tissue homeostasis and immune response to pathogens (6). These cells exhibit a high degree of plasticity in response to various external signals and are involved in both innate and adaptive immune responses; Under certain conditions, including stimulation by cytokines, macrophages are polarized into different phenotypes, classically activated macrophages (M1) and alternatively activated macrophages (M2) (7). However, recent research suggests that such traditionally nomenclature is too simplistic, as macrophages can express overlapping M1 and M2 gene (8). A better approach would be to describe it as a dynamic phenotypic spectrum, with M1 and M2 macrophages being the two extremes of this spectrum (9). Moreover, macrophages within the tumor microenvironment (known as tumor-associated macrophages) tend to be a different profile and their functional phenotypes are mainly determined by the surrounding context; therefore, TAMs are heterogeneous and exert a mixture of phenotypic characteristics, with not only the two extremes of a spectrum, namely M1 like macrophages and M2 like macrophages, but also other unknown polarized macrophage sets (10).

Previous evidences have suggested that massive infiltration of TAM or enrichment of TAM-related genes usually indicates tumor progression and a poor prognosis (11–13). In contrast, infiltration of TAM has also been found to predict a good prognosis in certain cancers, such as colorectal and ovarian carcinomas (14, 15). Indeed, TAMs play a dual role in tumors; they may exert pro-cancer effects *via* various pathways, such as by promoting tumor cell development, inducing tumor angiogenesis, promoting tumor cell metastasis and invasion, mediating resistance to drug therapy, and depressing anti-tumor immune responses (16). On the other hand, TAMs can be activated or reprogramed to trigger anti-tumor activities by secreting immunocidal molecules (e.g. ROS) and inflammatory cytokines (e.g. IFN-γ and TNF-α), or directly phagocytosing neoplastic cells and recruiting of tumor-killing leukocytes or activating adaptive immune responses (10, 17). Thus, these findings suggest that targeting TAMs and molecules associated with them could be a strategy for cancer treatment (16). In particular, the reprogramming of TAMs has attracted much attention. Here, we review the relationship between TAMs and cancer, discuss the origin, differentiation and phenotype of TAMs, and focus on the pro-tumor function of TAMs. Finally, we discuss therapies targeting TAMs, which are very promising strategies for cancer treatment.

## Molecular Characteristics of TAMs

### Origin of TAMs

**Figure 1:**
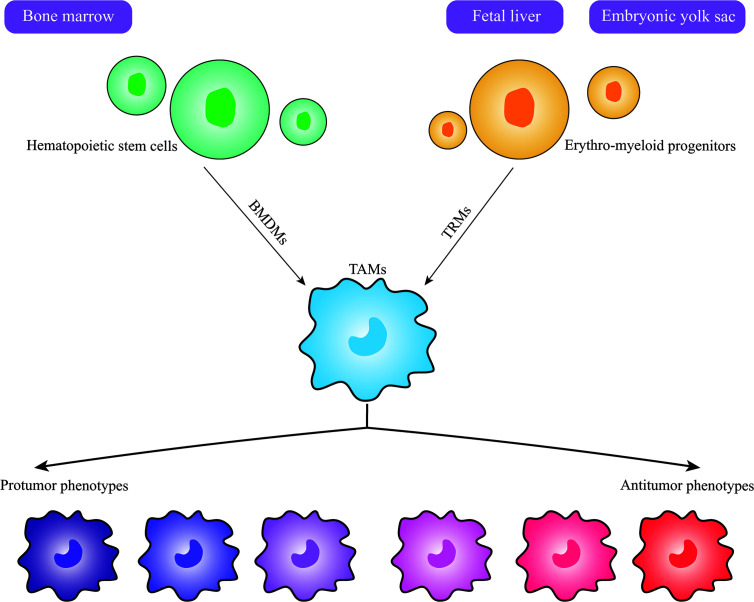
Origin and Heterogeneity of TAMs. Macrophages in tumors actually have at least three sources, including bone marrow, fetal liver and embryonic yolk sac. Bone-marrow-derived macrophages (BMDMs) develop from hematopoietic stem cells in the bone marrow, while tissue-resident macrophages (TRMs) develop from erythro-myeloid progenitors in the fetal liver or embryonic yolk sac. TAMs can differentiate into various phenotypes upon stimulation by different signals in the tumor microenvironment, and the protumor and antitumor phenotypes are the two extremes of the spectrum.

The origin of macrophages remains inconclusive. However, with the application of modern lineage tracing technology, the understanding of their origin has changed dramatically, macrophages actually derive from at least three sources: including the bone marrow (BM), the fetal liver and the embryonic yolk sac (16, 18). There are two general types of macrophages based on their origin and resident location, namely bone-marrow-derived macrophages (BMDMs) and tissue-resident macrophages (TRMs) (18). In general, hematopoietic stem cells (HSCs) in the bone marrow give rise to BMDMs, HSCs first develop into promonocytes and then enter the peripheral blood where they develop into mature monocytes, and finally exit capillaries and enter into the tissues to develop into tissue-specific macrophages (19). Nevertheless, the vast majority of TRMs originate from colony-stimulating factor 1 receptor (CSF1-R)-positive erythro-myeloid progenitors (EMPs) in the embryonic yolk sac or the fetal liver, which are different from BMDMs (18, 20). For a long time, it was believed that TRMs seeded in different tissue were gradually replenished and replaced by monocytes, which derived from HSCs in the adult BM. However, recent evidences indicate that TRMs are not replaced during embryonic development and that they proliferate locally and self-maintain independently throughout adulthood with little involvement of HSCs (21–23). In particular, microglia in the brain, a subset of TRMs, whose sole origin appears to be yolk sac-derived EMPs, seem never to be replaced by subsequently myeloid progenitors arising in fetal or adult life (23, 24). In tumor tissues, TAMs originate from either bone-marrow-derived macrophages or tissue-resident macrophages (**Figure 1**), but the proportion of TRM and BMDM in tumors varies considerably according to their tissue and organ specificity (25–27). Studies have reported that in most cases, TAMs largely originate from monocytes in the blood and are recruited into tumor tissue *via* chemokines (26). For example, in models of breast cancer, CCR2^+^ monocytes are the main source of TAMs, accounting for approximately 40% of all CD45^+^ cells within tumor tissues, compared to less than 10% derived from TRMs (26). In contrast, in pediatric solid tumors of fetal and postnatal developmental origin such as retinoblastoma, neuroblastoma, and osteosarcoma, TAMs are primarily derived from tissue-resident macrophages (25). Moreover, different sources of TAMs in the same tissue may exert different effects. For instance, in pancreatic ductal adenocarcinoma, TAMs are also mainly derived from embryonic-derived TRMs, which are more favorable for tumor cell proliferation and progression compared to monocyte-derived TAMs; in animal models, removal of tissue-resident macrophages can inhibit tumor progression (27). Another study on lung cancer indicates that monocyte-derived TAMs are correlate with promoting tumor spreading, while TRMs are associate with tumor growth *in vivo* (28). Therefore, these findings imply that both BMDMs and TRMs are present at different levels in different tumor models, and that the origin of TAMs has a potential influence on their functional changes, which suggests that targeting TAMs as a therapeutic strategy should be analyzed according to their origin and tissue organ and tumor specificity.

### Regulation of TAM Differentiation

Macrophages are regulated by a series of transcription factors during differentiation, among which PU.1 (also known as SPI1) plays a dominant role (29). However, macrophages from different tissues (e.g., lung, liver, or brain) exhibit a highly diverse transcriptional landscape (30). For instance, after *in-vivo* purified peritoneal macrophages were transplanted into the lungs of mice, they acquired a gene expression profile similar to that of alveolar macrophages (30). It was suggested that macrophage differentiation in tissues occurs locally and their regulation is driven to a large extent by the environment in which they reside. A similar situation may apply to TAMs, where the majority of TAMs in human tumors originate mainly from myeloid monocytes that are recruited to the tumor tissue and undergo a series of functional and phenotypic transformations to acquire TAM properties (31). There are also changes in expression of surface molecules, especially of mannose receptor (CD206), scavenger receptor (CD163) and arginase-1, which are expressed at higher levels (32). The transcriptional landscape of TAMs is not dependent on PU.1, but is regulated by signaling molecules from multiple sources, including signals form tumor and normal tissues; all off these signals must interact with developmental cues that control macrophage differentiation and ultimately mediate the properties and heterogeneity of TAMs (25). For example, in a mammary tumor model, unlike that of normal tissue macrophages, terminal differentiation of TAMs is dependent on activation of the Notch signaling pathway mediated by the transcriptional regulator RBPJ; furthermore, intervention in the Notch signaling pathway can have an anti-tumor effect by altering differentiation and maturation of TAMs (26). In contrast, knocking down RBPJ and thus interfering with the Notch signaling pathway not only failed to inhibit tumor growth, but also accelerated tumor proliferation in a hepatocellular carcinoma (33). In addition, the anti-cancer effect of type I interferon (IFN)-based therapies has been reported to target Ly6C^+^ monocytes and hinder their differentiation into TAMs (34). However, TAMs are well known for their plasticity, which allows them to acquire various phenotypes in different microenvironments. Thus, the applicability of TAM-targeted therapeutic strategies to other types of malignancies remains to be determined.

### Phenotypes and Function of TAMs

As an important class of innate immune cells, macrophages are recognized to be highly plastic and heterogeneous, and their phenotypes are regulated by the microenvironment in which they are located. For example, upon stimulation by proinflammatory factors such as lipopolysaccharide (LPS), IFN-γ, and tumor necrosis factor (TNF)-α, macrophages activate into M1-type macrophages (classical activation); while exposed to anti-inflammatory stimuli, including interleukin (IL)-4 and IL-13, they differentiated into M2-type macrophages (alternative activation) (7). When the body is invaded by pathogens, macrophages rapidly polarize into M1-type macrophages that secrete a variety of inflammatory factors (e.g., IL-6, IL-12, IL-1β and TNF-α) to promote an inflammatory response, engulf and destroy pathogens, process and present antigens, and initiate an adaptive immune response (7, 35). However, if the M1 phase becomes extended, it can cause tissue damage. Thus, macrophages polarize into the M2 type, releasing large amounts of anti-inflammatory cytokines (IL-10 and TGF-β) that attenuate an excessive inflammatory response and contribute to tissue repair, angiogenesis and maintenance of body homeostasis (36). It should be noted that M1 and M2 macrophages are merely the two extreme descriptions of the polarization state of macrophages and there are also other polarized macrophage populations (9). For instance, M2 macrophages can be induced to transform into M2a, M2b, M2c, and M2d subtypes under different conditions (37). M2a and M2c macrophages are functionally similar in terms of anti-inflammation, immunosuppression, and tissue repair, while M2b is more complex and associated with immune regulation; as for M2d macrophages, these cells are induced by co-stimulation by A2 adenosine receptor (A2R) and Toll-like receptor (TLR) agonists and are characterized by production of high levels of IL-10 and vascular endothelial growth factor (VEGF) and low levels of IL-12 and TNF-α (37–39). Some previous reviews had suggested that TAMs were equivalent to M2d macrophages (38). However, such classification of TAMs is difficult as this cell population is not a typical subgroup of macrophages and cannot be observed in the steady state, but is associated with specific pathological conditions, such as tumors and inflammation (40). Moreover, the phenotypic and functional heterogeneity of TAMs makes them highly variable between different tumors or even among different regions of the same tumor (41). Thus, the view that TAMs are equal to M2d macrophages is inaccurate.

Previous reports have indicated that TAMs can also be classified into M1-like macrophages (antitumor phenotype) and M2-like macrophages (protumor phenotype) (42, 43). Upon stimulation by proinflammatory factors (LPS, TNF-α), TAMs activate into M1-like macrophages and kill tumor cells by producing reactive oxygen species and inflammatory cytokines (e.g. IL-6 and TNF-α) (10). While in most types of tumor tissues, under the influence of signals originating from cancer cells or normal cells present in the TME, TAMs generally exhibit a higher degree of similarity to M2 macrophages and produce anti-inflammatory cytokines and chemokines (e.g. TGF-β, IL-10, CCL18, CCL22) that have little cytotoxic effect on tumor cells, but possess pro-tumor properties (16, 18). This is not only due to the overexpression of M2-type polarizing signals (such as IL-10, IL-4, IL-13, and TGF-β) in the TME, but is also related to the fact that pro-inflammatory cytokines can also confer pro-tumor properties to TAMs. For example, IFN-γ can directly enhance the expression of immunosuppressive enzymes such as indoleamine 2,3 dioxygenase (IDO) and nitric oxide synthase (NOS2) in TAMs, myeloid-derived suppressor cells (MDSCs), and dendritic cells (44, 45). Nevertheless, the M1 and M2 classification of TAMs mainly corresponds to *in vitro* conditions without environment influence, and TAMs *in vivo* are not so neatly divided into M1 and M2, especially within tumors (18). Thus, it is too simplistic to classify the phenotype and function of TAMs only from the perspective of M1 and M2 macrophages or based on a selection of markers. In addition, the gene signatures of TAMs in brain tumors are distinct from that of M1 and M2 *in vitro* polarized macrophages (46). Recent new technologies, such as single-cell RNA sequencing, multiplexed cytometry and mass cytometry by time-of-flight, also reveal that heterogeneity of TAMs may be more complex and diverse (47). Indeed, TAMs have various functional phenotypes and display remarkable plasticity and can play two opposing roles in cancer, including inhibiting and promoting tumor progression (**Figure 1**) (48). Additionally, TAMs have the ability to transform dynamically between antitumor and protumor phenotypes in respond to the flexible environment signals in the TME; this dramatically plasticity also leads to various subpopulation of TAMs (49). What’s more, several studies have reported that distribution of TAMs in the TME, such as inter- and intra-tumoral of TAMs, may have different phenotypes and functions in the same tumor (50). Therefore, TAMs may be more appropriately defined according to their ontogeny, activation status, function, intratumoral and intertumoral localization (49). Although TAMs are intricately heterogeneous and their role still needs further investigation, TAMs have profound effects in tumors and can be considered in cancer immunotherapy.

## Pro-Tumor Properties of TAMs

**Figure 2:**
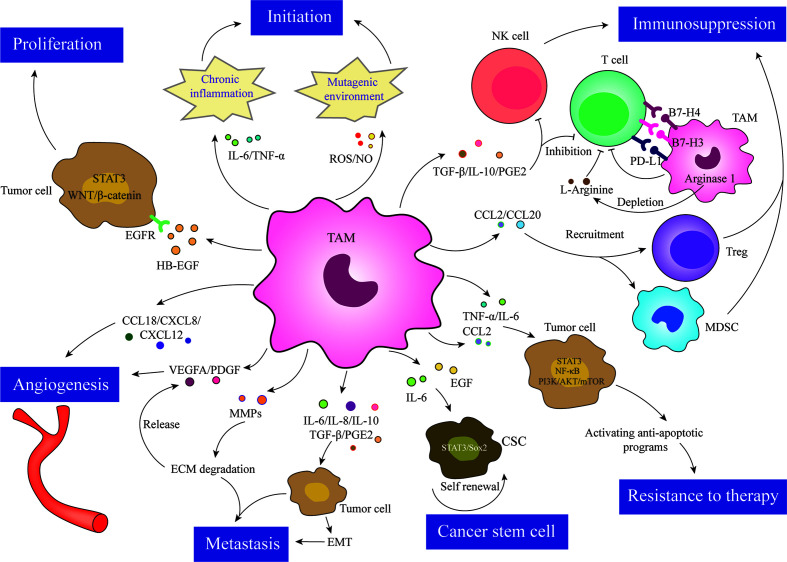
TAMs are involved in almost all aspects of tumor cell biology through various mechanisms, such as tumor initiation, proliferation, angiogenesis, metastasis, immunosuppression, resistance to therapy, and cancer stem cell maintenance.

Over recent decades, a plethora of studies have shown that TAMs exert pro-tumor effects and are closely associated with tumor progression. In fact, it has been demonstrated that TAMs exert their pro-cancer effects *via* two pathways: 1) direct pro-tumor properties, such as participating in the initiation of tumorigenesis, promoting tumor progression, metastasis to distant sites, and inducing therapeutic resistance in tumor cells, and 2) indirect pro-tumor properties, such as inhibiting anti-tumor immune responses to avoid damage to tumor cells and indirectly supporting tumor progression. A schematic depiction of the effects of TAMs in tumors is shown in **Figure 2**.

### TAMs in Tumor Initiation

During the 19^th^ century, it became apparent that a pathophysiological association between tumors and inflammation, especially long-term chronic inflammation, may contribute to initiation of cancers such as liver cancer, caused by the hepatitis B virus, and colon cancer, caused by inflammatory bowel disease (51, 52). Moreover, patients with chronic obstructive pulmonary disease (COPD), an inflammatory disease of the lungs, have an increased risk of lung carcinoma; bronchial inflammation induced by Haemophilus influenzae was also shown to increase the incidence of tumors in animal models of lung cancer (53, 54). In the microenvironment of chronic inflammation, there are a large number of inflammatory cells secreting various cytokines and growth factors, increasing the production of reactive substances, promoting oxidative DNA damage and reducing DNA repair, the net effect of which is a loss of normal cell damage control and repair. Thus, in a sense, tumors are like wounds that cannot be healed (55). Macrophages, as one of the most common immune cells in the tumor microenvironment, connect cancer and inflammation. During early carcinogenesis, TAMs are mainly characterized as proinflammatory macrophages, which induce the expression of inflammatory cytokines such as IL-6 and TNF-α; moreover, macrophages produce reactive oxygen and nitrogen species (ROS), and nitric oxide (NO) can react with peroxidase to produce nitrosoperoxycarbonate, further promoting inflammation and creating a mutagenic environment that causes mutations in normal epithelial cells (43). Furthermore, promoting inflammation by, e.g., silencing STAT3 in macrophages or inhibiting the expression of the anti-inflammatory factor IL-10, can further induce tumorigenesis (56, 57). By contrast, in a model of intestinal cancer, removal of ROS produced in macrophages suppresses carcinogenesis (58). After tumor formation, TAMs generally exhibit anti-inflammatory-associated markers such as arginase-1, CD206 and low levels of MHC-class II, and polarize into protumor macrophages that migrate into the tumor microenvironment, secreting growth factors that both suppress anti-tumor immune responses and support tumor cell proliferation (59). In mammary tumors, for example, TAMs secrete epidermal growth factor receptor (EGFR) family ligands, including heparin-binding EGF-like growth factor (HB-EGF), and activate STAT3-related signaling pathways to fuel tumor cell proliferation (60). Although not observed in all types of cancer, the pro-proliferative function of TAMs has been demonstrated in a variety of tumor studies, including pancreatic cancer, liver cancer, breast cancer, etc. (27, 33, 60, 61). For example, in hepatocellular carcinoma, blockade of the RBPJ-regulated Notch signaling pathway reduced infiltration of monocyte-derived TAMs into tumor tissues, but activated the WNT/β-catenin signaling pathway, leading to massive infiltration of Kupffer cell-derived TAMs into hepatocellular carcinoma tissues, which also upregulated anti-inflammatory cytokine IL-10 levels, downregulated IL-12 levels, and further facilitated tumor proliferation (33). In addition, it was shown that extracellularly regulated protein kinase 5 (ERK5) promoted macrophage polarization toward the protumor type, whereas silencing ERK5 expression in macrophages impaired STAT3 phosphorylation, induced TAM polarization toward the pro-inflammatory type, and exerted anti-tumor effects (61).

### TAMs in Tumor Progression

In addition to participating in tumor initiation and promotion, TAMs also play an important role in the progression from benign to malignant cancer. For example, in breast tumors, CSF-1 may accelerate tumor progression and the transition to malignancy by recruiting TAM infiltration (62). As with normal cells, tumor cells also require a vascular network to provide nutrients and oxygen and to remove metabolic waste for maintaining activity and metabolism (63). One of the key features of the progression of benign tumors to malignancy is overactive angiogenesis of the vascular network, a process that usually requires the involvement of innate immune cells; additionally, macrophages are important pro-angiogenic cells in the TME (64). Thus, TAMs support tumor angiogenesis mainly by the production of pro-angiogenic factors. Furthermore, cancer cells can also secrete large amounts of angiopoietin-2, which promotes the recruitment of monocytes expressing the angiopoietin 1 receptor (TIE2) to the tumor site, leading to massive infiltration of TAMs into the cancerous tissue (65). In addition to secreting pro-angiogenic growth factors [such as vascular endothelial growth factor A (VEGFA), platelet-derived growth factor (PDGF) and angiogenic chemokines (CCL18, CXCL8, CXCL12)], TAMs are also a major source of matrix metalloproteinase 9 (MMP-9), which facilitates extracellular matrix degradation and further contributes to the release of VEGFA (65, 66). Ultimately, the tumor microenvironment contains high levels of VEGF. In turn, VEGF recruits vascular endothelial cells and macrophages into the tumor tissue, contributing to abnormal tumor vascular formation, including excessive branching, vascular leakage and dead vessels, that together affect tumor hemodynamics and chemotherapeutic drug delivery (67). VEGF antagonists significantly reverse the abnormal vascular phenotype in tumors, induce vascular normalization and increase the sensitivity of tumor cells to chemotherapy (68). Moreover, studies have also shown that TAM-secreted CCL18 enhances vascular endothelial cell migration as well as angiogenesis. Further analysis revealed that CCL8 interacts with its receptor PITPNM3 on the surface of human umbilical vein endothelial cells (HUVECs) and activates ERK and AKT/GSK-3β/Snail signaling pathways in HUVECs, thereby contributing to its pro-angiogenic effects, while blockading PITPNM3 on the surface of HUVECs with neutralizing antibodies inhibits endothelial cell migration and angiogenesis (66). Other factors such as TGF-β, TNF, WNT7B, and thymidine phosphorylase promote tumor progression by recruiting and activating endothelial or other cells (such as fibroblasts) that further support angiogenesis in the tumor microenvironment (69, 70).

### TAMs in Tumor Metastasis

TAMs may trigger tumor initiation, accelerate tumor progression, and also contribute to cancer metastasis, leading to advanced stages of tumors. Tumor metastasis is characterized by cancer cells leaving the primary site and colonizing distant organs through blood or lymphatic vessels, which is one of the major causes of death in cancer patients (71). On the one hand, accumulating evidence has demonstrated that during metastasis, TAMs can induce epithelial–mesenchymal transitions (EMTs) in cancer cells, enhance tumor cell invasion, inhibit normal antigen presentation, reduce T cell recognition and destroy tumor cells by secreting various cytokines and inflammatory mediators such as IL-6, IL-8, IL-10, TGF-β and prostaglandin E2; on the other hand, TAMs also provide matrix remodeling enzymes and cathepsins that disrupt the tumor stroma by upregulating metalloproteinases such as MMP7 and MMP9, facilitating tumor cell migration away from the primary site (72, 73). In a model of colorectal cancer, for example, research has shown that TAMs were induced by tumor cells to secrete IL-6 that drove EMTs in tumor cells *via* the JAK2/STAT3/FoxQ1 axis, while in turn CCL2 produced by tumor cells recruited TAMs into the tumor microenvironment to form a vicious cycle that ultimately led to distant metastasis of colon cancer cells, and experiments *in vivo* revealed that inhibition of IL-6 significantly reduced tumor metastases (73). After leaving the primary site, tumor cells are attracted to blood vessels where they interact with perivascular TIE2^+^ macrophages, which increases vessel permeability and promotes tumor cell escape in part by secreting VEGFA (74). When invasive tumor cells enter the bloodstream, they need to survive in the blood or lymphatic circulation, avoid being recognized and eradicated by the immune system, reach distant organs and then colonize and grow in these normally hostile environments. Recent studies have shown that macrophages promote survival and growth of metastatic cancer cells by promoting the extravasation of invasive cancer cells from blood vessels, secreting signals that stimulate tumor cell growth and proliferation, and contributing to an immunosuppressive environment by suppression of cytotoxic T cell activity (75–77). Furthermore, it was found that CC-motif ligand 2 (CCL2) interacts with its receptor CCR2 to recruit inflammatory monocytes into the TME and become TAMs, which in turn can secrete another chemokine ligand, CCL3, to interact with metastatic cancer cells, improving TAM retention in metastatic foci and supporting tumor growth (76, 78). Moreover, in a lung metastasis model of breast cancer, metastatic cancer cells were observed to express vascular cell adhesion protein 1 (VCAM1) on their surface, which binds to α4-integrin, a molecular receptor on the surface of lung macrophages, which in turn activates the PI3K/Akt signaling pathway to maintain the survival of metastatic breast cancer cells in lung tissue (79).

### TAMs in Immunosuppression

TAMs may exert anti-neoplastic effects when they differentiate into proinflammatory phenotype. However, in most types of cancer in which signals originate from cancer cells or normal cells present in the TME, TAMs exhibit a higher degree of similarity to anti-inflammatory macrophages and skew TAMs to a pro-tumor state (16). TAMs can induce immunosuppression and contribute to tumor immune escape *via* multiple mechanisms. Numerous studies have shown that TAMs secrete cytokines such as TGF-β, IL-10, and PGE2 (prostaglandin E2), and that these immunosuppressive cytokines directly suppress effector functions of CD4^+^ and CD8^+^ T cells, increase Treg (regulatory T) cell expression and consequently contribute to an immunosuppressive microenvironment (80). In a model of breast cancer, for example, it was revealed that reducing PGE2 expression *via* deletion of cyclooxygenase-2 (COX-2) in TAMs could enhance T cell survival and immune surveillance and thereby suppress mammary tumor progression (81). Additionally, TAMs also cooperate with other immune cells to suppress anti-tumor immune responses. MDSCs (myeloid-derived suppressor cells) and Tregs are two types of cells in the tumor microenvironment that mediate immunosuppression. TAMs produce chemokines such as CCL2 and CCL20, which recruit these cells into the tumor microenvironment (82, 83). For example, CCL2 expressed by TAMs in gliomas is essential for recruitment of CCR4^+^ Tregs and CCR2^+^ Ly-6C^+^ MDSCs (82). Furthermore, research has indicated that expression of T cell immune checkpoint ligands (e.g., PD-L1) in TAMs may be an important TAM-mediated immunosuppression mechanism (84). For example, TAMs isolated from a mouse model of bladder cancer expressed high levels of PD-L1, suppressing tumor-specific T-cell immunity and enhancing tumor growth (84). Moreover, TAMs also express B7 family proteins such as B7-H3 and B7-H4, and inhibition of these proteins with antibodies impaired tumor progression in a CD8^+^ T and NK cell-mediated manner (85, 86). L-arginine is an essential amino acid for the CD3ζ chain in the T cell receptor complex that enhances the viability of activated T cells and the formation of memory T cells (87, 88). Expression of Arginase 1 on TAMs accelerates the metabolism of L-arginine to urea and L-ornithine, thereby further dampening T-cell recognition of tumor antigens and the antitumor immune response (48). In addition, TAMs also express inhibitory molecules, including non-classical major histocompatibility complex (MHC) class I molecules (e.g. HLA-E and HLA-G), which interact with inhibitory receptors (e.g. CD94 and leukocyte immunoglobulin-like receptor B subfamily member 1, LILRB1) on T and NK cells, to negatively regulate the activation of T and NK cells (89). Recent finding has implied that neoplastic cell can also express MHC class I molecules that interact with LILRB1 on TAMs and resist phagocytosis by TAMs, leading to loss of immune surveillance (90).

### TAMs in Other Pathways

Cancer stem cells (CSC), a class of tumor cells with a stemness phenotype, are capable of self-renewal and have multi-directional differentiation potential, and play an important role in tumor development, invasion, metastasis, and resistance to treatment (91). Similar to the microenvironment of tumor cells, cancer stem cells have their own unique microenvironment, or “niche”, consisting of fibroblasts and immune, perivascular and endothelial cells (92). TAMs, as key components of the CSC microenvironment, secrete cytokines and growth factors to support and promote the pro-cancer function of CSCs (93, 94). For example, research has shown that co-injection of CSCs and TAMs markedly increased tumor initiation activity and metastatic efficiency (93). Another study indicated that co-culture of TAMs with hepatocellular carcinoma stem cells increased the expansion of CSCs and promoted their incorporation into spheroids *in vitro* and xenograft tumors *in vivo*, while tocilizumab blocked IL-6 signaling and reduced their oncogenic potential (94). Moreover, it has been demonstrated that TAMs are involved in induction and maintenance of the CSC niche. It was suggested that EGF (epidermal growth factor) secreted by TAMs interacts with EGF receptors on breast cancer cells to further activate the STAT3/Sox2 signaling pathway to induce the CSC stemness phenotype (95). In addition to regulating CSC self-renewal and maintenance, TAMs can also induce therapeutic resistance by secreting growth factors and chemokines and by activating anti-apoptotic programs in cancer cells (67). For example, the sensitivity of breast cancer cells to tamoxifen was significantly reduced after culture in media that had been used for TAMs, and addition of CCL2 to the culture medium further reduced the sensitivity of tumor cells to tamoxifen. Further analysis revealed that CCL2 secreted by TAMs activated the PI3K/AKT/mTOR pathway in tumor cells, thus inducing resistance to tamoxifen treatment in breast cancer cells (96). Another study showed that combined treatment with CSF-1 antibodies and chemotherapeutic agents enhanced the sensitivity of breast cancer tumor cells to chemotherapy by reducing TAM recruitment to the tumor microenvironment and inhibiting the expression of multidrug resistance gene 1, chemoresistance genes and matrix metalloproteinases (97).

## Therapeutic Strategies Targeting TAMs

**Figure 3:**
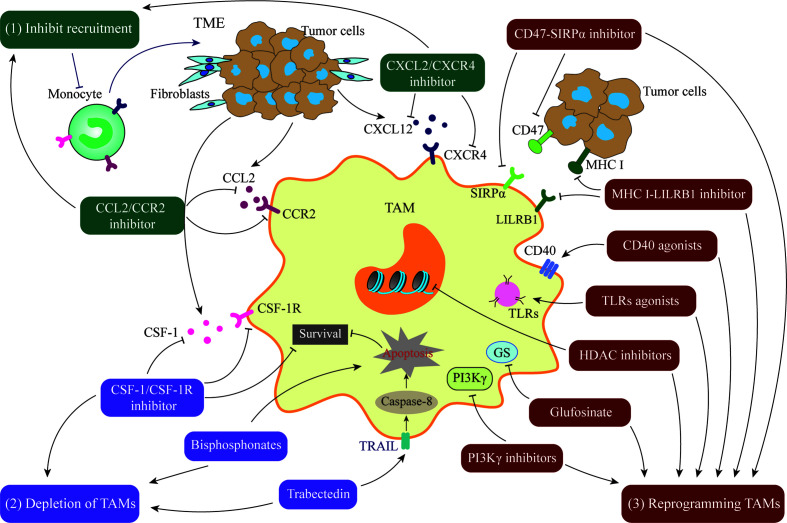
Treatment Strategies targeting TAMs. (1) Inhibition of TAM recruitment by blocking the CCL2/CCR2 or CXCL2/CXCR4 axis. (2) Depletion of TAMs by blocking the CSF-1/CSF-1R axis or using compounds such as bisphosphonates or trabectedin. (3) Reprogramming of TAMs to activate their anti-cancer function by CD47/STRPα pathway inhibitors, MHC I/LILRB1 pathway inhibitors, CD40 agonists, toll-like receptors agonists, Glufosinate, PI3Kγ inhibitors, or HDAC inhibitors.

Given the extensive involvement of TAMs in cancer cell biological processes, they are a potential target for cancer therapy. Currently, there are two main therapeutic strategies aimed at TAMs: (1) inhibition of TAM pro-cancer functions, including blockade of TAM recruitment or depletion of TAMs, and (2) “re-education” of TAMs to activate their anti-cancer function (**Figure 3**). Some drugs targeting TAMs in clinical trials are presented in **Table 1**.

**Table 1:** Examples of agents that target TAMs in clinical trials.

Treatment strategy	Agent name	Target	Drug type	Phase	Clinical trial number
Inhibit TAMs recruitment	Carlumab	CCL2	CCL2 antibody	II	NCT00992186
				I	NCT00537368
				Ib	NCT01204996
	MLN1202	CCR2	CCR2 antagonist	II	NCT01015560
	PF-04136309	CCR2	CCR2 antagonist	Ib	NCT01413022
	AZD5069	CCR2	CCR2 antagonist	I/II	NCT03177187
	CCX872-B	CCR2	CCR2 antagonist	Ib	NCT02345408
	Ulocuplumab	CXCR4	CXCR4 antibody	I	NCT01120457
	LY2510924	CXCR4	CXCR4 antibody	I	NCT02737072
	PTX-9908	CXCR4	CXCR4 antagonist	I/II	NCT03812874
	Motixafortide	CXCR4	CXCR4 antagonist	I/IIa	NCT01010880
				IIb	NCT02907099
Depletion of TAMs	MCS110	CSF-1	CSF-1 antibody	Ib/II	NCT02807844
				II	NCT03785496
	PLX3397	CSF-1R	CSF-1R inhibitor	I	NCT02777710
				Ib/II	NCT01596751
				III	NCT02371369
	DCC-3014	CSF-1R	CSF-1R inhibitor	I/II	NCT03069469
	BLZ945	CSF-1R	CSF-1R inhibitor	I/II	NCT02829723
	FPA008	CSF-1R	CSF-1R antibody	I	NCT03158272
				II	NCT02471716
	Bisphosphonates	NA	Small molecule	III	NCT00127205
				II	NCT00091832
	Trabectedin	Caspase 8	Small molecule	I	NCT03985722
				II	NCT02194231
Reprogramming TAMs	Hu5F9-G4	CD47	CD47 antibody	I	NCT02953782
	CC-95251	SIRPα	SIRP antibody	I	NCT03783403
	RO7009789	CD40	CD40 agonist	I	NCT02665416
	SEA-CD40	CD40	CD40 agonist	I	NCT02376699
	CP-870893	CD40	CD40 agonist	I	NCT01103635
	GSK1795091	TLR4	TLR agonist	I	NCT03447314
	IMO-2125	TLR7/8	TLR agonist	I	NCT03052205
				I/II	NCT02644967
				III	NCT03445533
	CMP-001	TLR9	TLR agonist	II	NCT03618641
	IPI-549	PI3Kγ	PI3Kγ inhibitor	Ib	NCT02637531
	YY-20394	PI3Kγ	PI3Kγ inhibitor	I	NCT03757000
	Chidamide	HDAC	HDAC inhibitor	II	NCT04512534

### Inhibiting Pro-Tumor TAMs

Blocking recruitment of macrophages into cancer tissue to reduce their pro-tumor effects is a promising therapeutic strategy. A plethora of cytokines and chemokines are involved in recruiting monocyte-derived macrophages into the tumor microenvironment, such as chemokine CCL2 (26, 76, 78, 96). Tumor cells and stromal cells in the tumor microenvironment can secrete CCL2, which interacts with its chemokine receptor CCR2 and exerts a critical role not only in recruiting bone marrow-derived monocytes into tumors and differentiation of monocytes into TAMs, but also in recruiting other immunosuppressive cells such as MDSCs and regulatory T cells (26, 76, 82). For example, in hepatocellular carcinoma, inhibition of CCL2 with specific monoclonal antibodies slows tumor progression and metastasis by blocking the recruitment of TAMs (78). Inactivation of neddylation was shown to significantly inhibit CCL2 secretion from lung cancer cells, reducing infiltration of monocytes into the tumor microenvironment and their development into TAMs, ultimately increasing survival (98). Thus, effective blockade of the CCL2/CCR2 axis is an effective way to restrict macrophage recruitment. At present, there are mainly two kinds of drugs being used in clinical trials: one is a CCL2-blocking antibody (carlumab, CNTO888), and the other is a small molecule inhibitor of CCR2 (PF-04136309) (16, 99). In a phase I clinical study, for example, the results showed that carlumab was well tolerated in patients with malignancy and exhibited some tumoricidal effects (99). However, a phase II clinical study in metastatic castration-resistant prostate cancer (mCRPC) revealed that carlumab did not restrain the CCL2/CCR2 pathway or exert antitumor effects, although the drug was also well tolerated in patients (100). The failure to restrict mCRPC progression suggests that blocking the CCL2/CCR2 axis in combination with other treatments might provide better anticancer effects. Indeed, overwhelming evidence supports this hypothesis. For example, in a study of pancreatic malignancy, it was shown that the CCR2 inhibitor PF-04136309 combined with FOLFIRINOX chemotherapy could achieve an objective tumor response and was safe and well tolerated (101). Furthermore, several studies have shown that the CXCL12/CXCR4 axis induces monocytes into the TME, which furthers tumorigenesis, and blockade of this axis can hinder TAM recruitment and accumulation (102–104). For instance, a study revealed that the CXCR4 antagonist, BL-8040, inhibits neuroblastoma tumors and has therapeutic potential in pediatric cancer (105). Another study indicated that the CXCL12 peptide antagonist, CTCE-99088, markedly slows tumor growth and reduces distant metastases in breast cancer (106). In addition, it was showed that chemokine CX3CL1 interacts with its receptor CX3CR1 to induce TAM recruitment into tumor tissues and promote skin cancer progression (107). Therefore, CX3CL1/CX3CR1-mediated signaling may be a new potential target for therapies that inhibit TAM recruitment into the tumor microenvironment.

In addition to elimination of TAMs by blocking macrophage recruitment, depletion of TAMs, e.g., by induction of TAM apoptosis, has also received much attention. Colony-stimulating factor 1 receptor (CSF-1R), a member of the tyrosine kinase receptor family, binds to its ligands CSF-1 or IL-34 and triggers homodimerization of the receptor and subsequent activation of receptor signaling (107). CSF-1/CSF-1R-mediated signaling plays a critical role in the survival, differentiation and maturation of macrophages (16). Numerous studies have reported that high expression of CSF-1 or its receptor CSF-1R is associated with poor prognosis in malignant tumors, such as in breast cancer and Hodgkin’s lymphoma (62, 97, 108). Furthermore, CSF-1/CSF-1R signaling contributes to the conversion of TAMs from a tumor-suppressor phenotype to a tumor-promoter phenotype (109). Thus, blocking CSF-1 and CSF-1R-mediated signaling can be a promising strategy for cancer immunotherapy. Currently, several small molecules aimed at CSF-1/CSF-1R are being tested in clinical studies, including PLX3397/Pexidartinib, DCC-3014, BLZ945, FPA008/Cabiralizumab, and MCS110 (110). For instance, a study showed that PLX3397, an antagonist of CSF-1R, inhibited cell proliferation in gliomas and induced tumor regression *via* depletion of TAMs (111). Another study indicated that after PLX3397 treatment in mice with breast cancer, there was a significant depletion of macrophages in tumor tissues accompanied by an increase in the ratio of CD8^+^/CD4^+^ T cells and significantly reduced tumor growth (112). Moreover, it was demonstrated that inhibition of CSF-1R can improve the prognosis of cancer patients (109, 112). Tenosynovial giant cell tumors (TGCTs), for example, are a rare and locally aggressive type of tumor characterized by high levels of CSF-1 and overexpression of CSF-1R^+^ macrophages (113, 114). In a clinical phase III trial for this disease, patients treated with pexidartinib showed significant improvement in symptoms and functional outcomes, and thus pexidartinib could be a potential treatment for patients with TGCT (114). However, a clinical phase II study in recurrent glioblastoma indicated that CSF-1R inhibition with PLX3397 did not significantly improve progression free survival (PFS) and failed to increase overall survival, although the agent avidly crossed the blood-tumor barrier and was well tolerated in patients (115). Other clinical data did not show significant antitumor activity of CSF1R inhibitors against relapsed or refractory classical Hodgkin lymphoma (116), implying limited clinical efficacy of targeting CSF-1 or CSF-1R and leading some companies to discontinue their myeloid targeting programs. It was observed that infiltration of MDSCs in tumor tissues increased after CSF-1R blockade, which may explain, to some extent, the limitations of the clinical efficacy of CSF-1 or CSF-1R inhibitors (117). Collectively, blockade of the CSF-1/CSF-1R axis may be a potential strategy in cancer therapy, especially for TGCTs. Nevertheless, it is increasingly clear that selecting the right patient based on tumor type, in combination with other appropriate treatments, may provide clinical benefit to cancer patients.

In addition, numerous studies have reported that some compounds, such as bisphosphonates and trabectedin, effectively remove macrophages from the tumor microenvironment by inducing apoptosis of macrophages (118, 119). Bisphosphonates are structurally stable, similar to pyrophosphatase, and can interact with osteoclasts to prevent bone resorption and bone metastasis (120). In breast tumors, for instance, it was observed that pyrophosphatase could be taken up by TAMs, in which it interferes with multiple functions, including polarization and survival, to exert anti-neoplastic effects (121). Trabectedin, an antineoplastic drug, could induce death of monocytes/macrophages *via* a TNF-related apoptosis-inducing ligand (TRAIL)-mediated mechanism (122). A study indicated that trabectedin restricts melanoma growth and metastasis *via* reducing the number of TAMs in the tumor microenvironment (123).

### Activating Anti-Tumor TAMs

Although a plethora of preclinical and clinical studies have revealed that elimination of TAMs can be an effective tumor treatment, we hypothesize that not all macrophages will be depleted. In addition, pro-inflammatory and tumoricidal macrophages will still be present in the TME, and if all macrophages were eliminated, the organism would potentially be at risk when encountering infectious diseases. Moreover, macrophages are well known for their plasticity in which they acquire various phenotypes, such as proinflammatory and anti-inflammatory types, in different microenvironments. Thus, reprogramming TAMs to activate pro-inflammatory, anti-tumor properties may lead to more effective cancer treatments. In the following we summarize strategies for “re-educating” TAMs to exert anti-neoplastic effects.

Myeloid cells, such as dendritic cells and macrophages, express signal-regulated protein alpha (SIRPα), a protein that recognizes and binds to CD47 molecules (124). CD47 is expressed on both normal and cancer cells, and interacts with SIRPα expressed on macrophages to send a “don’t eat me” signal, thus avoiding phagocytosis by macrophages (125). Hence, it is possible to restore TAM recognition and phagocytosis of tumor cells and to activate anti-neoplastic immune responses by interfering with the SIRPα-CD47 axis (e.g., by using antibodies to SIRPα and CD47) (126). This strategy was used in a study of glioblastoma, where it was observed that a CD47 monoclonal antibody converted tumor-promoting TAMs to an anti-tumor phenotype that exhibited enhanced phagocytosis of cancer cells (127). Another study found that antibody blockade of CD47 significantly increased phagocytosis of hepatocellular carcinoma cells by macrophages and promoted infiltration of proinflammatory macrophages into tumor tissue to further eliminate tumor cells (128). Moreover, it was shown that the humanized anti-CD47 antibody Hu5F9-G4 could block recognition and interaction of CD47-SIRPα, but only slightly inhibited the activity of normal human neuronal cells (129). Therefore Hu5F9-G4 is promising for the safe treatment of malignant pediatric brain tumors.

Moreover, recent evidences have indicated that there are other “don’t eat me” signals, such as the MHC class I-LILRB1 axis (90, 130, 131). LILRB, a subfamily of leukocyte immunoglobulin-like receptor (LILR) family, contains five members (LILRB1–5), is a class of inhibitory receptors with intracellular immunoreceptor tyrosine-based inhibitory motifs (ITIM) (132). MHC class I is expressed in many nucleated cells including tumor cells, and its β2-microglobulin subunit interacts with LILRB1, which is wildly expressed on monocytes and TAMs, thereby protecting cancer cells from being engulfed by macrophages (90, 130). Furthermore, blocking both CD47 and MHC I produces a synergistic effect and a stronger tumor suppression, which indicate CD47 and MHC I signals may work cooperatively (90). However, antitumor activity of cytotoxic T cells depends on the antigen presentation of MHC. Thus, strategies that specifically block β2-microglobulin or LILRB1 seem to be more promising. LILRB2, another best-characterized member of LILRB family, is also mostly expressed on macrophages, monocytes and CD4^+^ T cells (131). It was suggested that LILRB2 exerted a critical role in the maturation of macrophages; antagonism of LILRB2 with specific monoclonal antibodies could impair the inhibitory effect of macrophages on T cell proliferation, reduce the infiltration of MDSCs and Tregs in tumor tissue, enhance the phagocytosis by macrophages, reprogram TAMs to a proinflammatory phenotype and promote antitumor immunity (133). Additionally, recent study reported that blockade of LILRB4 could convert anti-inflammatory macrophages to a more inflammatory phenotype, which may be another potential target for therapies that activate anti-cancer function of TAMs (134). However, these mechanisms need to be further confirmed and validated in more clinical trials.

In addition to blocking “don’t eat me” signals, there are other strategies that target TAMs, such as CD40 agonists, toll-like receptor agonists, PI3Kγ inhibitors, and histone deacetylase (HDAC) inhibitors (10, 16). CD40, a molecule of the TNF receptor superfamily, is expressed on macrophages and other antigen-presenting cells (APCs). Once activated by its ligand CD40L, CD40 promotes the expression of MHC molecules and stimulates the release of pro-inflammatory cytokines from APCs, which exert anti-neoplastic effects by supporting effector T-cells (135). In a study of pancreatic ductal adenocarcinoma, for example, it was observed that a CD40 agonist prompted macrophage infiltration into tumor tissue and converted TAMs to an anti−tumor phenotype, and a combination of CD40 agonists and the chemotherapeutic agent gemcitabine resulted in tumor remission in patients with advanced disease (136). In addition, it has been shown that CD40 agonists exhibit more potent antitumor efficacy when combined with mitogen-activated protein kinase inhibitors (MEKi) by enhancing tumoricidal immune responses and attenuating the immunosuppressive cell activities of M2 macrophages, Tregs and MDSCs (137).

Toll-like receptors (TLRs) are an evolutionarily ancient family of pattern recognition receptors that play a key role in activating the innate immune response; TLRs can induce macrophage differentiation into a pro-inflammatory phenotype upon activation by viral nucleic acids (i.e., DNA or RNA) or bacterial particles (e.g., lipopolysaccharides) (138). Based on this property of TLRs, TLR agonists, such as TLR4, TLR7/8 and TLR9, are currently tested in cancer research with some success in stimulating TAM polarization into an anti-tumor phenotype (139). For instance, it was observed that the TLR7/8 agonist can induce MDSCs to differentiate into tumoricidal macrophages, which makes it a potential agent for treatment of oxaliplatin-resistant colorectal carcinoma (140). Notably, bacilli calmette guerin (BCG), the first FDA-approved TLR-agonist for the treatment of bladder cancer, stimulates TLR2 and TLR4, which convert TAMs toward anti−tumor types and enhance the cytotoxicity of macrophages against neoplastic cells (141). Furthermore, intratumoral administration of TLR-stimulating drugs has not only shown effective local antitumor efficacy, but also reduces distant tumor metastasis by activating the systemic immune system (142). Additionally, TLR7/8 agonist MEDI9197 combined with immune checkpoint inhibitors PD-1 blockade can polarize TAMs toward anti-tumor phenotypes and activate CD8^+^ T cells and NK cells, leading to a better efficacy (143).

In addition, a number of kinase, metabolic or epigenetic enzyme inhibitors are being tested *in vitro* and *in vivo* to control the polarization and activation of macrophages (144). For example, PI3Kγ is widely expressed on macrophages and other myeloid cells, and is the only class 1B PI3K member (145). Activation of PI3Kγ signaling can trigger an immunosuppressive transcriptional profile of TAMs and promote cancer progression (146). As a result, PI3Kγ inactivation induces tumor regression *via* up-regulation of pro-inflammatory cytokines such as IL-12 and stimulation of CD8^+^ T cells (146). Glutamine not only plays a critical role in numerous biological functions (e.g., amino acid production, nucleotide synthesis, and extracellular matrix production), but also promotes tumor growth and creates an immunosuppressive TME (147). However, Inhibition of glutamine synthetase (GS) with an antagonist, glufosinate, can impair cancer metastasis in several mouse tumor models, which is associated with blockade of immunosuppression and angiogenesis and re-education of TAMs to an anti-neoplastic type (148). Additionally, studies have reported that epigenetic remodeling of TAMs, such as inhibition of histone deacetylase (HDAC), promotes repolarization of macrophages to an anti-tumor phenotype and activates T cell responses (149, 150). Moreover, research has shown that TMP195, an antagonist of HDAC, reduced tumor growth and metastasis by modulating TAMs into a tumoricidal phenotype; furthermore, TMP195 enhanced the antitumor effect of PD-1 treatment and chemotherapy (150). Collectively, these results demonstrate that reprogramming TAMs to anti−tumorigenic macrophages is a promising strategy that has the potential to improve the prognosis of cancer patients.

As mentioned above, the manipulation of TAMs to exert anti-neoplastic effects has been investigated using different therapeutic approaches, such as CD47-SIRP1a or MHC I- LILRB1inhibitors can restore the ability of TAMs to engulf tumor cells, TLRs and CD40 agonists and other inhibitors can stimulate TAMs to become tumoricidal effector cells. All of these reprogramming strategies are based on activated macrophages that are either directly cytotoxic to tumor cells or indirectly activate cytotoxic T cells or NK cells. However, there is evidence suggests that anti-tumor TAMs are required for efficacy of therapy response. In a study of pancreatic ductal adenocarcinoma, patients with substantial infiltration of TAMs at the tumor site responded better to gemcitabine; further analysis indicates that TAMs exposed to gemcitabine were converted to antitumor types with activation of cytotoxicity genes (151). Similarly, paclitaxel, a chemotherapeutic drug, is showed to rewire TAMs toward tumoricidal phenotypes through activation of TLR4 on TAMs (152). Another clinical phase II trial in metastatic gastric cancer suggested that high infiltration of M2 macrophages in the TME may enhance the sensitivity of cabazitaxel (a novel taxoid) and improve survival (153). The mechanism by which TAMs enhance the efficacy of chemotherapy may be related to the immunogenic cell death (ICD) of neoplastic cells caused by chemotherapeutic drugs (154). When tumor cells die through ICD, they may emit a large number of molecules, such as damage-associated molecular patterns (DAMP), cytokines, tumor antigens and immunostimulatory signals, which ultimately switch TAMs toward tumoricidal phenotypes and activate adaptive immune responses (155). Other therapies such as radiotherapy, and cancer vaccines, may also require TAMs to enhance efficacy (156, 157). In addition to being activated by ICD death of tumor cells, TAMs can also be induced and activated by cytotoxic T cells to exert anti-tumor functions after therapy. In turn, tumoricidal TAMs possess the ability to support effector T cells. For example, in a model of cervical carcinoma, synthetic long peptide (SLP) vaccines can induce tumor regression not only by inducing cytotoxic CD8+ T cells to infiltrate tumor sites, but also by attracting macrophages by these vaccine-induced T cells; depletion of TAMs with CSF-1R inhibitor abrogates the complete tumor remission elicited by SLP therapy (157). Another study suggests that specific depletion of the CD163+ macrophages can trigger tumor shrinkage in a melanoma model by increasing infiltration of effector T cells and concomitant recruitment of pro-inflammatory TAMs (158). Likewise, it is implied that IL-1β contributes to an immunosuppressive TME and favors tumor growth; inhibition of IL1β drives the accumulation of CD8^+^ T cells at tumor sites, which subsequently activates TAMs and induces tumor regression; nevertheless, depletion of TAMs with CSF-1R inhibitor can abolish tumor shrinkage caused by IL-1β deficient (159). These findings suggest that macrophage-T cell cross-talk plays an important role in the anti-tumor immune response. However, more research is needed to better understand these mechanisms.

## Conclusion

TAMs, an important class of innate immune cells in the tumor microenvironment, are widely expressed in a variety of tumor tissues, while massive infiltration of TAMs or enrichment of TAM-related genes usually indicates tumor progression or poor disease prognosis. Moreover, studies have overwhelmingly shown that TAMs are intimately involved in multiple tumor-related processes by contributing to tumorigenesis and proliferation, accelerating angiogenesis, promoting invasion and metastasis, inducing cancer stem cell formation, triggering treatment resistance, and immunosuppression. Thus, the various tumor-promoting mechanisms of TAMs exhibit numerous appealing targets for cancer treatment. Tumor immunotherapies targeting TAMs, including inhibition of TAM recruitment, acceleration of TAM depletion or apoptosis, activation of phagocytosis by TAMs, and modulation of TAM anti−tumor polarization, have shown great potential in preclinical and clinical cancer research. It is encouraging to see that drugs targeting TAMs are in clinical trials and showing anti-tumor effects. Collectively, strategies that target TAMs may well turn out to be promising in tumor immunotherapy.

Nevertheless, there are some issues and limitations that remain to be addressed. Firstly, TRM-derived TAMs and monocyte-derived TAMs are both present in the TME. Recruitment of monocyte-derived TAMs can be achieved by inhibition of trafficking receptors (e.g., CC2R), but TRMs do not seem to be recruited in this way. Thus, strategies that can differentially block the recruitment of TRMs are needed. Secondly, non-specific depletion of TAMs results in some tumoricidal macrophages also being eliminated. Therefore, the long-term consequences of TAM depletion are still unclear. Finally, repolarization of TAMs towards an anti-tumorigenic phenotype may trigger excessive macrophage activation and thus be associated with substantial toxicity, such as macrophage activation syndrome and hemophagocytic syndrome. Therefore, more research is needed to deepen the understanding of TAMs.

## Author Contributions

YT conceived and designed the figure, as well as wrote and revised the manuscript. MW collected the related reference and revised the manuscript. YZ and SG edited the manuscript. FZ, GX, and CS provided guidance and revised the manuscript. All authors contributed to the article and approved the submitted version.

## Funding

This work was supported by the National Key Research and Development Program of China (2018YFC0910700, 2016YFB0201702) and Chenguang Program of Shanghai Municipal Education Commission (No.158554).

## Conflict of Interest

The authors declare that the research was conducted in the absence of any commercial or financial relationships that could be construed as a potential conflict of interest.

## Glossary

A2R, A2 adenosine receptor; APCs, antigen-presenting cells; BCG, bacilli calmette Guerin; BM, bone marrow; BMDMs, bone-marrow-derived macrophages; CSF-1, colony-stimulating factor 1; CSF1-R, CSF-1 receptor; CD206, mannose receptor, CD163, scavenger receptor; COPD, chronic obstructive pulmonary disease; CCL, chemokine (C-C motif) ligand; CCR, chemokine (C-C motif) receptor; CXCL, CXC motif chemokine ligand; CXCR: CXC chemokine receptor; CTLs, cytotoxic CD8+ T lymphocytes; COX-2, cyclooxygenase-2; CSCs, cancer stem cells; CX3CL1, C-X3-C motif chemokine ligand 1; CX3CR1, CX3C chemokine ligand 1 receptor; DAMP, damage-associated molecular patterns; DCs, dendritic cells; EMPs, erythro-myeloid progenitors; EGF, epidermal growth factor; HB-EGF, heparin-binding EGF; EGFR, EGF receptor; ERK5, extracellularly regulated protein kinase 5; EMT, epithelial-mesenchymal transition; ECM, extracellular matrix; GS, glutamine synthetase; HSCs, hematopoietic stem cells; HUVECs, human umbilical vein endothelial cells; HDAC, histone deacetylase; HLA, human leukocyte antigen; ICD, immunogenic cell death; IL, interleukin; IFN-γ, interferon-γ; IDO, indoleamine 2,3 dioxygenase; Igs, immunoglobulins; LPS, lipopolysaccharide; ITIM, immunoreceptor tyrosine-based inhibitory motifs; LILR, leukocyte immunoglobulin-like receptor; LILRB1, leukocyte immunoglobulin-like receptor B subfamily member 1; MDSCs, myeloid-derived suppressor cells; MMPs, matrix metalloproteinases; MHC, major histocompatibility complex; MHC I, major histocompatibility complex class I; mCRPC, metastatic castration-resistant prostate cancer; MEKi, mitogen-activated protein kinase inhibitors; NO, nitric oxide; NOS2, nitric oxide synthase; NK cells, Natural killer cells; NF-κB, nuclear factor kappa B; PDGF, platelet-derived growth factor; PEG2, prostaglandin-E2; PFS, progression free survival; ROS, reactive oxygen species; STAT3, Signal transducer and activator of transcription 3; SIRPα, signal-regulated protein alpha; TAMs, tumor-associated macrophages; TME, tumor microenvironment; TRMs, tissue-resident macrophages; TNF-α, tumor necrosis factor-α; TLRs, Toll-like receptors; TIE2, angiopoietin 1 receptor; Tregs, regulatory T cells; TRAIL, TNF-related apoptosis-inducing ligand; TGCT, Tenosynovial giant cell tumor; VEGF, vascular endothelial growth factor; VEGF-A, vascular endothelial growth factor A; VCAM1, vascular cell adhesion protein 1.
